# Supplementary material for: Quantum Annealing in the NISQ Era: Railway Conflict Management
Source: Entropy (Basel). 2023 Jan 18;25(2):191. doi: 10.3390/e25020191 (PMC9955039; doi:10.3390/e25020191)
Supplement: Supplementary file 1 [file entropy-25-00191-s001.zip › entropy-2101138-supplementary.pdf]

Quantum annealing in the NISQ era: railway conflict management
SUPPLEMENTAL MATERIAL

Krzysztof Domino,[1, *] Mátyás Koniorczyk,[2, †] Krzysztof Krawiec,[3, ‡]
Konrad Jałowiecki,[4, §] Sebastian Deffner,[5, ¶] and Bartłomiej Gardas[1, **]

[1] Institute of Theoretical and Applied Informatics, Polish Academy of Sciences, Bałtycka 5, 44-100 Gliwice, Poland
[2] Wigner Research Centre, Konkoly-Thege M. út 29-33, H-1525 Budapest, Hungary
[3] Faculty of Transport and Aviation Engineering, Silesian University of Technology, 40-019 Katowice, Poland
[4] Institute of Physics, University of Silesia, 41-500 Chorzów, Poland;
[5] Department of Physics, University of Maryland, Baltimore County, Baltimore, MD 21250, USA; and Instituto de Física 'Gleb Wataghin', Universidade Estadual de Campinas, Campinas 13083-859, SP, Brazil

(Dated: January 17, 2023)

The present supplemental material contains a technically detailed description our models as well as additional examples of solutions, obtained with heuristics, CPLEX's QUBO solver, and tensor networks. In particular, in Section SI we describe the model's complete notation and its constraints in a way common in the Operations Research literature. Section SII is devoted to a linear integer programming formulation of the model which is used for a comparison with a classical approach. Section SIII provides all the details of the quadratic unconstrained binary (QUBO) formulation. I Section SIV we comment further on classical algorithms for solving QUBO (or Ising) problems. Finally, Section SV contains further computational results concerning particular railway situations.

SI. OUR MODEL: DETAILS

In this section, we introduce the model of the railway line and the dispatching conditions. Table I provides a comprehensive summary of the notation used.

A. Railway line

We assume a railway line $\mathcal{M}$ to be a set of block sections: segments that can be occupied by at most one train at a time, as discussed in the paper. These are either *line blocks* or *station blocks*; both are also refereed to as *block sections* or just *blocks*. The set of line blocks are denoted by $\mathcal{L}$, and the set of station blocks by $\mathcal{S}$. This model also incorporates sidings or double-track sections by treating them as station blocks.

Trains can only meet and pass (M-P) or meet and overtake (M-O) at stations. We follow the buffer approach by treating each station as a block that can be occupied by up to b trains at a time, where b is the number of tracks at the station. The other *blocks* can be occupied by only one train at a time.

The set of trains is denoted by $\mathcal{J}$ and is split into the subset of trains traveling in a given direction $\mathcal{J}^0$ and the subset of trains going in the opposite direction $\mathcal{J}^1$:

$$\mathcal{J}^0 \cup \mathcal{J}^1 = \mathcal{J} \text{ and } \mathcal{J}^0 \cap \mathcal{J}^1 = \emptyset. \tag{S1}$$

Let $j \in \mathcal{J}$ be a particular train. Its route is a sequence of *blocks* $M_j = (m_{j,1}, m_{j,2}, \ldots, m_{j,\text{end}})$, where $m_{j,1}$ is the starting block and $m_{j,\text{end}}$ is the ending block. Each block (from M_j) is passed by train j once and only once (i.e., we do not consider recirculation). Given a train j and a block $m_{j,k}$, the preceding block is $\pi_j(m_{j,k}) = m_{j,k-1}$, while the subsequent block is $\rho_j(m_{j,k}) = m_{j,k+1}$. We assume that neither $\rho_j(m_{j,\text{end}})$ nor $\pi_j(m_{j,1})$ belongs to the analyzed *network* segment.

We assume that a route can be defined solely by a sequence of station blocks $S_j = (s_{j,1}, s_{j,2}, \ldots, s_{j,\text{end}})$, where M-P and M-O may occur (i.e., there are no alternative routes between stations). Similarly to *blocks* in general, for a train j and a station block $s_{j,k}$, we denote the preceding station block as $\pi_j(s_{j,k}) = s_{j,k-1}$, and the subsequent station

* kdomino@iitis.pl
† koniorczyk.matyas@wigner.hu
‡ krzysztof.krawiec@polsl.pl
§ dexter2206@gmail.com
¶ deffner@umbc.edu
** bartek.gardas@gmail.com

symbol	description / explanation
$A_{j,s}$	discretized set of all possible delays of train j at station s
$\mathcal{H}(t)$, $\mathcal{H}_0$, $\mathcal{H}_p$	time-dependent Hamiltonian of the annealing process and its time-independent components
$t \in [0, T]$	quantum annealing time
$\hat{\sigma}^x, \hat{\sigma}^z$	Pauli matrices
$j \in \mathcal{J}$	trains (jobs)
$\mathcal{J}^0$ ($\mathcal{J}^1$)	trains heading in a given (opposite) direction
$m \in \mathcal{M}$	*blocks* (machines)
$s \in \mathcal{S}$	station blocks
$l \in \mathcal{L}$	line blocks
$M_j, (S_j)$	the sequence of *blocks* (station blocks) in the route of j
$s_{j,1}, s_{j,k}, s_{j,\text{end}}$	the first, k-th, and last station block in the route of j
$m_{j,1}, m_{j,k}, m_{j,\text{end}}$	the first, k-th, and last block in the route of j
$S_j = (s_{j,1}, s_{j,2}, \ldots, s_{j,\text{end}})$	a sequence of all station blocks in j's route
S_j^*, (S_j^{**})	a sequence of station blocks in j's route without the last (last two) elements
$S_{j,j'}$	a common path of j and j', ordered according to j's path
$S_{j,j'}^*$	a common path of j and j' excluding the last block, ordered according to j's path
$\rho_j(m), \rho_j(s)$	the subsequent block (station block) in j's route
$\pi_j(m), \pi_j(s)$	the preceding block (station block) in j 's route
$t_{\text{out}}(j,s)$, ($t_{\text{in}}(j,s)$)	time of leaving (entering) station block s by train j
$t_{\text{out}}^{\text{timetable}}(j,s)$	timetable time of leaving s by j
$p_{\text{timetable}}(j,m)$, $p_{\min}(j,m)$	timetable and minimum time of j passing m
$d(j,s)$	delay of j leaving s
$d_U(j,s)$	primary (unavoidable) delay of j leaving s
$d_s(j,s)$	secondary delay of j leaving s
$d_{\max}(j)$	maximum possible (acceptable) secondary delay for train j
$\tau_{(1)}(j,...)\tau_{(2)}(j,...)$	minimum time for train j to give way to another train going in the same (opposite) direction
$x, (\mathbf{x})$	binary decision variable (vector of binary decision variables), e.g., $x_{j,s,d} = x_i$ is 1 if j leaves s with a delay d and 0 otherwise, $i \in \{1, 2, \ldots, n\}$
$Q \in \mathbb{R}^{n \times n}$	symmetric QUBO matrix, where n is the number of logical quantum bits.
$f(\mathbf{x})$	objective function; the weighted sum of secondary delays

TABLE I: Notation summary

block as $\rho_j(s_{j,k}) = s_{j,k+1}$. It is convenient to assume that all train paths start and end at stations; hence we have $s_{j,1} = m_{j,1}$ and $s_{j,\text{end}} = m_{j,\text{end}}$.

B. Delay representation

Ideally, the time $t_{\text{out}}(j,s)$ when train j leaves station block s should be the time prescribed by the timetable, $t_{\text{out}}^{\text{timetable}}(j,s)$. If, however, $t_{\text{out}}(j,s) > t_{\text{out}}^{\text{timetable}}(j,s)$, there is a delay in leaving station block s:

$$d(j,s) = t_{\text{out}}(j,s) - t_{\text{out}}^{\text{timetable}}(j,s). \tag{S2}$$

Primary or unavoidable delays (as defined in Section II A) are denoted by $d_U(j,s)$. If an already delayed train enters a railway line $\mathcal{M}$, the initial delay will appear at the first block $d_U(j, s_{j,1})$. The unavoidable delay propagates along the line, thereby providing a lower bound of the overall delay. Unavoidable delays are non-negative, so we have

$$d_U(j, \rho_j(s)) = \max\{d_U(j,s) - \alpha(j,s,\rho_j(s)), 0\}, \tag{S3}$$

where $\alpha(j,s,\rho_j(s))$ accounts for the possible time reserve in passing the sequence of *blocks*, starting from the one directly after s and ending at station block $\rho_j(s)$. In the same way, the unavoidable delays are propagated due to the minimum times of the rolling stock circulation at terminals. Importantly, all unavoidable delays can be computed prior to the optimization.

The secondary delay $d_S(j,s)$ is denoted by

$$d_S(j,s) = d(j,s) - d_U(j,s). \tag{S4}$$

We introduce upper bounds $d_{\max}(j)$ of the secondary delays as parameters of the model. Their values can either be determined manually (maximum acceptable secondary delays of the given trains) or be obtained by using some fast heuristics such as the first come first served (FCFS) or first leave first served (FLFS) approach (c.f. Section IV B of the paper). Setting them too low, however, can result in an unfeasible model.

Having established the upper and lower bounds,

$$d_U(j,s) \leq d(j,s) \leq d_U(j,s) + d_{\max}(j), \tag{S5}$$

we can use the (integer) values of the delays as decision variables. The bounds ensure that these variables remain in a finite range. In what follows, we shall call this description, in terms of the discretized delays as decision variables, "delay representation"; it will be very convenient from the QUBO modeling point of view.

C. Dispatching conditions

Consider a train j whose path M_j consists of both station and line blocks. We assume that the leaving time of the given block equals the entering time of the subsequent block:

$$t_{\text{out}}(j,m) = t_{\text{in}}(j, \rho_j(m)). \tag{S6}$$

(This is a slight simplification as there is a finite time in which the train is located in both *blocks.*) For each train $j \in \mathcal{J}$ and each block $m \in M_j$, two kinds of passing times are assigned: a nominal (timetable) $p_{\text{timetable}}(j,m)$ and a minimum $p_{\min}(j,m)$. Note that the latter can be smaller or equal to $p_{\text{timetable}}(j,m)$ (as there can be a reserve).

We address common dispatching conditions, including: the minimum passing time condition, the single block occupation condition, the deadlock condition, the rolling stock circulation condition at the terminal, and the capacity condition.

Condition SI.1. The minimum passing time condition. The leaving time from the block section cannot be lower than the sum of the entering time and the minimum passing time:

$$t_{\text{out}}(j,m) \geq t_{\text{in}}(j,m) + p_{\min}(j,m). \tag{S7}$$

For subsequent station blocks $s = m_{j,k}$ and $\rho_j(s) = m_{j,l}$, we have

$$t_{\text{out}}(j, \rho_j(s)) \geq t_{\text{out}}(j,s) + \sum_{i=k+1}^{l} p_{\min}(j, m_{j,i}) = t_{\text{out}}(j,s) + \sum_{i=k+1}^{l} p_{\text{timetable}}(j, m_i) - \alpha(j, s, \rho_j(s)), \tag{S8}$$

where $\alpha(j,s,\rho_j(s))$ is the time reserve mentioned before. In the delay representation, this condition takes the simple form

$$d(j, \rho_j(s)) \geq d(j,s) - \alpha(j,s,\rho_j(s)). \tag{S9}$$

(Compare this with Eq. (S3), where we have an equal sign for the lower limit.)

Condition SI.2. The single block occupation condition. Let j and j' be two trains heading in the same direction and sharing their routes between station s and subsequent station $\rho_j(s)$. If train j leaves station block s at time $t_{\text{out}}(j,s)$, the subsequent ($t_{\text{out}}(j',s) \geq t_{\text{out}}(j,s)$) train j' can leave this block at a time for which the following equation is fulfilled:

$$t_{\text{out}}(j',s) \geq t_{\text{out}}(j,s) + \tau_{(1)}(j,s,\rho_j(s)), \tag{S10}$$

where $\tau_{(1)}(j,s,\rho_j(s))$ is the time required for train j to give way to train j' on the route between station block s and subsequent station block $\rho_j(s)$. In the delay representation we have:

$$d(j',s) + t_{\text{out}}^{\text{timetable}}(j',s) \geq d(j,s) + t_{\text{out}}^{\text{timetable}}(j,s) + \tau_{(1)}(j,s,\rho_j(s)) \tag{S11}$$

or

$$d(j', s) \geq d(j, s) + t_{\text{out}}^{\text{timetable}}(j, s) - t_{\text{out}}^{\text{timetable}}(j', s) + \tau_{(1)}(j, s, \rho_j(s)). \tag{S12}$$

Hence, taking $\Delta(j, s, j', s) = t_{\text{out}}^{\text{timetable}}(j, s) - t_{\text{out}}^{\text{timetable}}(j', s)$, we get

$$d(j', s) \geq d(j, s) + \Delta(j, s, j', s) + \tau_{(1)}(j, s, \rho_j(s)). \tag{S13}$$

As mentioned before, the condition in Eq. (S13) needs to be tested for $t_{\text{out}}(j', s) \geq t_{\text{out}}(j, s)$, i.e., $d(j', s) \geq d(j, s) + \Delta(j, s, j', s)$; otherwise trains must be investigated in the reversed order.

The actual form of $\tau_{(1)}(j, s, \rho_j(s))$ depends on the dispatching details of the particular problem. We assume that all the time reserves are realized on stations. Consequently, $\tau_{(1)}(j, s, \rho_j(s))$ is delay independent, which makes the problem tractable.

Condition SI.3. The deadlock condition. Assume that two trains j and j' are heading in opposite directions on a route determined by subsequent station blocks s and $\rho_j(s)$ in the path of train j. In the path of j', these are reversed, so j goes $s \to \rho_j(s)$, while j' goes $\rho_j(s) \to s$. Assume for now that the train j will enter the common block section before j'. (This condition must also be checked in the reverse order.) Let $\tau_{(2)}(j, s, \rho_j(s))$ be the time required for train j to get from station block s to $\rho_j(s)$. Given this, the deadlock condition can be stated as follows:

$$t_{\text{out}}(j', \rho_j(s)) \geq t_{\text{in}}(j, \rho_j(s)), \tag{S14}$$

i.e.,

$$t_{\text{out}}(j', \rho_j(s)) \geq t_{\text{out}}(j, s) + \tau_{(2)}(j, s, \rho_j(s)). \tag{S15}$$

In the delay representation,

$$d(j', \rho_j(s)) + t_{\text{out}}^{\text{timetable}}(j', \rho_j(s)) \geq d(j, s) + t_{\text{out}}^{\text{timetable}}(j, s) + \tau_{(2)}(j, s, \rho_j(s)) \tag{S16}$$

and

$$d(j', \rho_j(s)) \geq d(j, s) + t_{\text{out}}^{\text{timetable}}(j, s) - t_{\text{out}}^{\text{timetable}}(j', \rho_j(s)) + \tau_{(2)}(j, s, \rho_j(s)). \tag{S17}$$

Hence, taking $\Delta(j, s, j', \rho_j(s)) = t_{\text{out}}^{\text{timetable}}(j, s) - t_{\text{out}}^{\text{timetable}}(j', \rho_j(s))$, we get:

$$d(j', \rho_j(s)) \geq d(j, s) + \Delta(j, s, j', \rho_j(s)) + \tau_{(2)}(j, s, \rho_j(s)). \tag{S18}$$

Again, condition Eq. (S18) needs to be tested for $t_{\text{out}}(j', \rho_j(s)) \geq t_{\text{out}}(j, s)$; otherwise trains must be investigated in the reversed order.

Further, similarly to Condition SI.2, the form of $\tau_{(2)}(j, s, \rho_j(s))$ depends on the dispatching details resulting from the formulation of the problem. Again, as all time reserves are assumed to be realized at stations, $\tau_{(2)}(j, s, \rho_j(s))$ is delay independent, which makes the problem more tractable.

As mentioned before, the particular form of the τ-s are problem dependent; we propose the following approach to this. Suppose that train j departs from station s to subsequent station $\rho_j(s)$, passing the *blocks* $m_k, m_{k+1}, \ldots, m_{l-1}, m_l$, where $s = m_k$ and $m_l = \rho_j(s)$. The subsequent train proceeding in the same direction is allowed to leave at least after

$$\tau_{(1)}(j, s) = \max_{i \in \{k+1, \ldots, l-1\}} \left(t_{\text{in}}^{\text{timetable}}(j, m_{i+1}) - t_{\text{in}}^{\text{timetable}}(j, m_i) \right). \tag{S19}$$

The subsequent train proceeding in the opposite direction is allowed to leave at least after

$$\tau_{(2)}(j, s) = \sum_{i \in \{k+1, \ldots, l-1\}} \left(t_{\text{in}}^{\text{timetable}}(j, m_{i+1}) - t_{\text{in}}^{\text{timetable}}(j, m_i) \right) \equiv t_{\text{in}}^{\text{timetable}}(j, \rho_j(s)) - t_{\text{out}}^{\text{timetable}}(j, s). \tag{S20}$$

Referring to the minimum and maximum delay conditions – see Eq. (S5) – there are pairs of trains for which either Condition SI.2, or Condition SI.3, is always fulfilled. This observation simplifies our QUBO representation of the problem.

Condition SI.4. Rolling stock circulation condition at the terminal. If train j with a given train set assigned terminates at a station where the next train j' of the same train set starts its course (after turnover), i.e., $s_{j,end} = s_{1,j'}$, the following condition arises:

$$t_{\text{out}}(j', s_{j',1}) > t_{\text{in}}(j, s_{j,end}) + \Delta(j, j'), \tag{S21}$$

where $\Delta(j, j')$ is the minimum turnover time. In the delay representation, we have

$$d(j', 1) + t_{\text{out}}^{\text{timetable}}(j', 1) > d(j, s_{j,end-1}) + t_{\text{out}}^{\text{timetable}}(j, s_{j,end-1}) + \tau_{(2)}\left(j, s_{j,end-1})\right) + \Delta(j, j'). \tag{S22}$$

Hence, taking $R(j, j') = t_{\text{out}}^{\text{timetable}}(j', 1) - t_{\text{out}}^{\text{timetable}}(j, s_{j,end-1}) - \tau_{(2)}\left(j, s_{j,end-1})\right) - \Delta(j, j')$, we get

$$d(j', 1) > d(j, s_{j,end-1}) - R(j, j'). \tag{S23}$$

Condition SI.5. The capacity condition. Here we include the buffer approach of handling stations in our model. Suppose we have a station block s, capable of handling up to b trains at a time. Let $\{j_1, j_2, \ldots, j_{b+1}\} \subset \mathcal{J}$ be any $b+1$-tuple of trains. No time t may exist for which all the conditions below are simultaneously fulfilled:

$$\begin{aligned} t_{\text{in}}(j_1, s) \quad &\leq t \leq t_{\text{out}}(j_1, s) \\ &\ldots \\ t_{\text{in}}(j_{b+1}, s) &\leq t \leq t_{\text{out}}(j_{b+1}, s). \end{aligned} \tag{S24}$$

In the delay representation,

$$\begin{aligned} d(j_1, \pi_{j_1}(s)) + t_{\text{out}}^{\text{timetable}}(j_1, \pi_{j_1}(s)) + \tau_{(2)}\left(j_1, \pi_j(s)\right) \quad &\leq t \leq d(j_1, s) + t_{\text{out}}^{\text{timetable}}(j_1, s) \\ &\ldots \\ d(j_{b+1}, \pi_{j_1}(s)) + t_{\text{out}}^{\text{timetable}}(j_{b+1}, \pi_{j_{b+1}}(s)) + \tau_{(2)}\left(j_{b+1}, \pi_j(s)\right) &\leq t \leq d(j_{b+1}, s) + t_{\text{out}}^{\text{timetable}}(j_{b+1}, s). \end{aligned} \tag{S25}$$

As a consequence of Condition SI.5, many new constraints may arise. These may make the calculations more complex, even exceeding the capacity of the current quantum computers. In our particular problem instances, we will temporarily ignore this condition, but we will verify the solutions against it.

Finally, it is worth observing that Conditions SI.1 - SI.5 refer to station blocks only; line blocks do not appear. As we have a single-track line, there is no need to analyze line blocks in the optimization algorithm: the decisions are made at the stations. The leaving time from the ending (station) block does not have to be analyzed either.

SII. LINEAR INTEGER PROGRAMMING APPROACH

Before proceeding towards the QUBO approach we introduce a linear integer programming formulation, too. This does not needed to get a QUBO model, however, it is in the line with the standard treatment of railway dispatching problems. Meanwhile it is formulated so that it is compares easily with the QUBO approach. It will therefore be used as a reference for comparisons.

Similarly to the model in [1], we opt for using precedence variables as it is very suitable for a single-track railway model. We introduce the binary decision variables $y_{j,j',k}$ so that they have a value of 1 if the train j occupies the particular part of the track (denoted by k) before train j', and are zero otherwise.

Train delays will be represented with discrete decision variables $d(j, s)$ that fulfil Eq. (S5). (The discretization is not necessary, but it is practical for the comparison with the QUBO results, as the discretization is required there and our particular problem instances were found to be tractable with a standard solver.)

Note that the ordering of the train departures is uniquely described by the precedence variables (y-s), but for each configuration there is still some freedom in determining the value of the delay variables (d-s). For the solution to be valid, the values of the y-s and d-s should be consistent; this will be ensured by the constraints.

The constraints are the following. The constraints in Eq. (S9), and Eq. (S23) are linear; hence, they can directly be included in the model. The single block occupation condition, see Eq. (S13), is expressed in terms of the precedence and delay variables:

$$d(j', s) + M \cdot (1 - y_{j,j',s}) \geq d(j, s) + \Delta(j, s, j's) + \tau_{(1)}(j, s, \rho_j(s)), \tag{S26}$$

where $y_{j,j',s}$ determines the order of trains j and j' leaving station s, and M is an arbitrary large number. For two trains j and j' heading in opposite directions, the deadlock condition is to be prescribed. For trains with a common path between subsequent stations $k \to \{s, \rho_j(s)\}$, the requirement in Eq. (S18) takes the following form:

$$d(j', s) + M \cdot (1 - y_{j,j',k}) \geq d(j, s) + \Delta(j, s, j'\rho_j(s)) + \tau_{(2)}(j, s, \rho_j(s)), \tag{S27}$$

where $y_{j,j',k}$ determines which train enters the common path first.

Finally, as to the objective function, the weighted sum of secondary delays (or the total weighted tardiness in the scheduling terminology) will be minimized, which is also inherently linear:

$$\min \sum_j \frac{d(j, s_{j,\text{end}-1}) - d_U(j, s_{j,\text{end}-1})}{d_{\max}(j)} w_j, \tag{S28}$$

where w_j is the weight reflecting the train's priority. Having formulated a linear model of the problem for comparison, let us now return to the QUBO model.

SIII. QUBO FORMULATION OF OUR MODEL

We construct a QUBO model that can be solved either by quantum annealers or by classical algorithms inspired by them. After presenting a constrained 0-1 representation, we employ a penalty method to move the constraints to the effective objective function to get an unconstrained problem. This is maybe the most challenging step, not only in our present work, but also in logical programming using QUBOs.

A. 0-1 program representation

As a step toward a QUBO model, we formulate our problem entirely in terms of binary decision variables. We achieve this by the discretization of time, i.e., the discretization of the delay variables. Hence, we need to set a delay resolution step. We opt for a resolution of one minute as this is reasonable from train timetabling point of view (and the generalization is straightforward). Given such a representation, Eq. (S5) can be rewritten into the following form:

$$d(j, s) \in A_{j,s} = \{d_U(j, s), d_U(j, s) + 1, \ldots, d_U(j, s) + d_{\max}(j)\}, \tag{S29}$$

where $A_{j,s}$ is a discretized set of all possible delays of train j at station s.

For the QUBO representation, we introduce the binary decision variables

$$x_{s,j,d} \in \{0, 1\}, \tag{S30}$$

which take the value of 1 if train j leaves station block s at delay d, and zero otherwise. These variables will also be referred to as "QUBO variables." Their vector is $\mathbf{x} \in \{0, 1\}^n$. Each variable is assigned a logical quantum bit. Hence solving the problem requires n of these bits. The number n depends on the size of the system and is dependent on the number of trains and stations and the value of the maximum secondary delay.

We assume that each train leaves each station block once and only once:

$$\forall_j \forall_{s \in S_j} \sum_{d \in A_{j,s}} x_{s,j,d} = 1. \tag{S31}$$

Remark SIII.1. Observe that Conditions SI.2 and SI.3 (the single block occupation condition and the deadlock condition) refer to the subsequent stations in train j path – s and $\rho_j(s)$. (Recall that $\rho_j(s_{j,\text{end}})$ does not exist in our model.) Time of entering of $\rho_j(s)$ is computed from $x_{s,j,d}$ and $\tau_{(1)}(j, s, \rho_j(s))$, but it does not refer to $x_{\rho_j(s),j,d}$. Hence we do not need to investigate the leaving time from the last block of the train's path. We assume that the arrival time at this block can be computed from the leaving time from the penultimate block and the passing time. (Of course, our goal is to reduce the number of QUBO variables in the analysis.) Here, delays at the end of the route are investigated on leaving the penultimate station of the analyzed route.

Let $S_{j,j'}$ be the sequence of *blocks* in the common route of trains j and j'. If both these trains are traveling in the same direction, the order of *blocks* in $S_{j,j'}$ is straightforward. Alternatively, we need to regard the block sequence of train j as the reversed sequence of *blocks* of train j'. Therefore, we introduce $S^*_{j,j'} = S_{j,j'} \setminus \{s_{j,\text{end}}\}$ for Conditions SI.2 and SI.3. Condition SI.2 states that two trains traveling in the same direction are not allowed to appear at the same block section. In particular, from Eq. (S13) it follows that

$$\forall_{(j,j') \in \mathcal{J}^0(\mathcal{J}^1)} \forall_{s \in S^*_{j,j'}} \sum_{d \in A_{j,s}} \left(\sum_{d' \in B(d) \cap A_{j',s}} x_{j,s,d} x_{j',s,d'} \right) = 0, \tag{S32}$$

where $B(d) = \{d + \Delta(j, s, j', s), d + \Delta(j, s, j', s) + 1, \ldots, d + \Delta(j, s, j', s) + \tau_{(1)}(j, s, \rho_j(s)) - 1\}$ is a set of delays that violates Condition SI.2.

Assume now that two trains j and j' are heading in opposite directions. From Eq. (S18) it follows that

$$\forall_{j\in\mathcal{J}^0(\mathcal{J}^1), j'\in\mathcal{J}^1(\mathcal{J}^0)} \forall_{s\in S^*_{j,j'}} \sum_{d\in A_{j,s}} \left(\sum_{d'\in C(d)\cap A_{j',\rho_j(s)}} x_{j,s,d} x_{j',\rho_j(s),d'} \right) = 0 \tag{S33}$$

where $C(d) = \{d(j,s)+\Delta(j,s,j',\rho_j(s)), d(j,s)+\Delta(j,s,j',\rho_j(s))+1, \ldots, d(j,s)+\Delta(j,s,j',\rho_j(s))+\tau_{(2)}(j,s,\rho_j(s))-1\}$.

We do not need to examine delays when leaving the ending station of the train's path; see Remark SIII.1. For the minimum passing time – Condition SI.1 – we introduce $S^{**}_j = S_j \setminus \{s_{j,end}, s_{j,end-1}\}$. From Eq. (S9) we have:

$$\forall_j \forall_{s\in S^{**}_j} \sum_{d\in A_{j,s}} \left(\sum_{d'\in D(d)\cap A_{j,\rho_j(s)}} x_{j,s,d} x_{j,\rho_j(s),d'} \right) = 0, \tag{S34}$$

where $D(d) = \{0, 1, \ldots, d - \alpha(j, s, \rho_j(s)) - 1\}$.

Following the the rolling stock circulation (Condition SI.4) we have, from Eq. (S23),

$$\forall_{j,j'\in\text{terminal pairs}} \sum_{d\in A_{j,s_{(j,end-1)}}} \sum_{d'\in E(d)\cap A_{j',1}} x_{j,s_{(j,end-1)},d} \cdot x_{j',s_{(j,'1)},d'} = 0, \tag{S35}$$

where $E(d) = \{0, 1, \ldots, d - R(j, j')\}$.

The objective of the algorithm is to schedule trains so that secondary delays are minimized. The general objective function can be written in the following form:

$$f(d, j, s) = \hat{f}\left(\hat{d}, j, s\right), \tag{S36}$$

where $\hat{d} = \frac{d(j,s)-d_U(j,s)}{d_{\max}(j)}$. As discussed in Section II A, *primary delays* (d_U) are unavoidable, so they are not relevant for the objective. Recall that upper bounds of the secondary delays $d_{\max}(j)$ have been introduced as parameters, see Eq. (S5). Thus we require $\hat{f}(\hat{d}, j, s)$ to obey the following conditions:

$$\hat{f}(\hat{d}, j, s) = \begin{cases} 0 & \text{if } \hat{d} = 0, \\ \max_{\hat{d}\in[0,1]} \hat{f}(\hat{d}, j, s) & \text{if } \hat{d} = 1, \\ \text{is non-decreasing in } \hat{d} & \text{if } \hat{d} \in (0, 1). \end{cases} \tag{S37}$$

This non-decreasing property reflects that higher delays cannot contribute to a lower extent to the objective. Finally, our objective function will be linear:

$$f(\mathbf{x}) = \sum_{j\in J} \sum_{s\in S^*_j} \sum_{d\in A_{j,s}} f(d, j, s) \cdot x_{j,s,d}, \tag{S38}$$

where $f(d, j, s)$ are the weights.

Apart from the constraints discussed above, the penalty function can be chosen deliberately, which adds some relevant flexibility to the model. By selecting the appropriate $\hat{f}(\hat{d}, j, s)$, various dispatching policies can be represented. This ensures freedom of choice in striving for the best suited dispatching solution. Let us mention just a few of them:

1. For a quasi-minimization of the maximum secondary delays, one may opt for a strongly increasing convex function in $\hat{d}$, such as an exponential or geometrical.
2. To minimize the number of delayed trains, one may opt for the step function $\hat{d}$.
3. To minimize the sum of delays, one may opt for a linear function in $\hat{d}$.
4. Subsequent trains can be assigned various weights for the delays on which their priorities depend.
5. A subset of stations can be selected as the only relevant stations from the point of view of delays. For practical reasons, we analyze delays on penultimate stations – see Remark SIII.1.

For our particular dispatching problems, we select the policies set out in Points 3 – 5.

B. A remark on the penalty coefficients

To get some hint of how to determine the coefficients of the summands that warrant feasibility, let us first consider a direct search solution of a QUBO of the form in Eq. (4). This amounts to evaluating the objective function with all possible values of the decision variables. In our effective QUBO in Eq. (26), the total matrix Q is a sum of the terms in Eq. (22) and Eq. (25) and the original objective function of Eq. (20). So we have a sum of three QUBOs, and the objective function value is linear in the matrix of QUBOs. Hence, the objective value will be the sum of the original objective function value and the values of the summands representing the constraints.

The feasibility terms have a negative minimum $-L$ because of the omitted 0th order terms when using Eq. (25) (instead of Eq. (24) if the solution is feasible). For each element of the outer sum in Eq. (25), the value p_{sum} contributes to L, hence $L = p_{\text{sum}} \cdot$ (the number of linear constraints). The value

$$f''(\mathbf{x}) = \mathcal{P}_{\text{pair}} + \mathcal{P}_{\text{sum}} - L \tag{S39}$$

will be zero if the solution is feasible, and non-zero otherwise. We will call it the hard constraints' penalty.

If there is solution in which the "cost" of violating some hard constraints is lower than the particular objective function value, the effective QUBO may yield a minimum that is unfeasible. A way to avoid this is to ensure that the lowest violation of any hard constraint has a larger contribution to $f'(\mathbf{x})$ than a violation of all soft constraints (encoded in the objective $f(\mathbf{x})$) of a given feasible (not necessarily optimal) solution. Such a solution can be obtained by some fast heuristics.

This suggests that one should assign high coefficients to the hard constraints. If one employs a direct search algorithm calculating the values of the objective very accurately, this approach can work out easily. However, the numerical accuracy is always limited, and other inaccuracies of the minimum search can also appear. In the case of a quantum annealer, this is due to the noise of the system. What we get in reality is not the guaranteed to be absolute minimum but a set of samples: vectors for which the effective objective function is close to the minimum. If the coefficients are too high, the original objective function is just a small perturbation over the feasibility violations. Hence, while obtaining strictly feasible solutions, the actual minimum can be lost in the noise. Therefore finding the appropriate values of p_{sum} and p_{pair} amounts to finding the values that address both the criteria of both feasibility and optimality to a suitable extent.

C. A simple example

Let us demonstrate our approach in a simple example. Consider two trains $j \in \{1, 2\}$, two stations $s \in \{1, 2\}$, and a single track between them. The passing time value (scheduled and minimum) between the stations is 1 (minute) for both trains. Train $j = 1$ is ready to depart from station $s = 1$ (heading to $s = 2$) at the same time as train $j = 2$ is ready to depart from station $s = 2$ (heading to $s = 1$). Under these circumstances, a conflict appears on a single track between the stations.

Let the initial delay of both trains be $d = d_U = 1$. As one of the trains needs to wait a minute to meet and pass the other one, the maximum acceptable secondary delay is $d_{\text{max}} = 1$; see Eq. (S29). Taking the QUBO representation as in Eq. (S30) (i.e., $x_{s,j,d}$), we have the following 4 quantum bits: $x_{1,1,1}, x_{1,1,2}$ (train 1 can leave station 1 at delay 1 or 2), $x_{2,2,1}$, and $x_{2,2,2}$ (train 2 can leave station 2 at delay 1 or 2). The linear constraints express that each train departs from each station once and only once, so Eq. (S31) takes the form

$$x_{1,1,1} + x_{1,1,2} = 1 \text{ and } x_{2,2,1} + x_{2,2,2} = 1. \tag{S40}$$

Referring to Eq. (25), the optimization subproblem is as follows:

$$\mathcal{P}_{\text{sum}} = -p_{\text{sum}} \left(x_{1,1,1}^2 + x_{1,1,2}^2 - x_{1,1,1}x_{1,1,2} - x_{1,1,2}x_{1,1,1} + x_{2,2,1}^2 + x_{2,2,2}^2 - x_{2,2,1}x_{2,2,2} - x_{2,2,2}x_{2,2,1} \right), \tag{S41}$$

with the optimal value equal to $-L = -2p_{\text{sum}}$.

The quadratic constraint is that the two trains are not allowed to depart from the stations at the same time, i.e., $x_{1,1,1}x_{2,2,1} = 0$ and $x_{1,1,2}x_{2,2,2} = 0$. Using Eq. (22), the optimization subproblem takes the following form:

$$\mathcal{P}_{\text{pair}} = p_{\text{pair}} \left(x_{1,1,1}x_{2,2,1} + x_{2,2,1}x_{1,1,1} + x_{1,1,2}x_{2,2,2} + x_{2,2,2}x_{1,1,2} \right), \tag{S42}$$

with the optimal value equal to 0. Note that since we have only two stations in this simple example, the minimum passing time condition does not appear ($S^{**} = \emptyset$).

Finally, a possible objective function is

$$f(\mathbf{x}) = x_{1,1,2}w_1 + x_{2,2,2}w_2 = x_{1,1,2}^2 w_1 + x_{2,2,2}^2 w_2, \tag{S43}$$

where the secondary delay of train 1 is penalized by w_1 and the secondary delay of train 2 is penalized by w_2.

Let the vector of decision variables be denoted by $\mathbf{x} = [x_{1,1,1}, x_{1,1,2}, x_{2,2,1}, x_{2,2,2}]^T$. The QUBO problem can thus be written in the form of Eq. (4), so

$$Q = \begin{bmatrix} -p_{\text{sum}} & p_{\text{sum}} & p_{\text{pair}} & 0 \\ p_{\text{sum}} & -p_{\text{sum}} + w_1 & 0 & p_{\text{pair}} \\ p_{\text{pair}} & 0 & -p_{\text{sum}} & p_{\text{sum}} \\ 0 & p_{\text{pair}} & p_{\text{sum}} & -p_{\text{sum}} + w_2 \end{bmatrix}. \tag{S44}$$

As the solution is parameter dependent, we can use various trains prioritization policies. For the sake of demonstration, assume that train $j = 2$ is assigned a higher priority than train $j = 1$. This implies the assignment of different penalty weights. We set $w_1 = 0.5$ and $w_2 = 1$.

As discussed in Section III C, to ensure that the calculated solution is feasible, we require that the following conditions are met: $p_{\text{sum}} > \max\{w_1, w_2\}$ and $p_{\text{pair}} > \max\{w_1, w_2\}$. We propose $p_{\text{pair}} = p_{\text{sum}} = 1.75$, so matrix Q to takes following form:

$$Q = \begin{bmatrix} -1.75 & 1.75 & 1.75 & 0 \\ 1.75 & -1.25 & 0 & 1.75 \\ 1.75 & 0 & -1.75 & 1.75 \\ 0 & 1.75 & 1.75 & -0.75 \end{bmatrix}. \tag{S45}$$

The optimal solution is $\mathbf{x} = [0, 1, 1, 0]^T$ (train 2 goes first) with $f'(\mathbf{x}) = -3$. Another feasible solution (not optimal) is $\mathbf{x} = [1, 0, 0, 1]^T$ (train 1 goes first) with $f'(\mathbf{x}) = -2.5$. The other solutions are not feasible: for example, $\mathbf{x} = [1, 0, 1, 0]^T$ is not feasible as the two trains are expected to depart from the stations at the same time, with $f'(\mathbf{x}) = 0$. Observe that the classical heuristics (such as FCFS and FLFS) do not make a difference between the two feasible solutions, as both trains enter the conflict segment at the same time and need the same time to pass it. Also, both solutions have the same value of the secondary delay.

Having formulated our model as a QUBO problem, it is ready to be solved on a physical quantum annealer or by a suitable algorithm.

SIV. CLASSICAL ALGORITHMS FOR SOLVING ISING PROBLEMS

An additional benefit of formulating problems in terms of Ising-type models is that the existing methods developed in statistical and solid-state physics for finding ground states of physical systems can also be used to solve an Ising-type model or, equivalently, a QUBO model on classical hardware. Notably, variational methods based on finitely correlated states (such as matrix product states for 1D systems or projected entangled pair states suitable for 2D graphs) have had a very extensive development in the past few decades. A quantum information theoretic insight into density matrix renormalization group methods (DMRG [2]) – being the most powerful numerical techniques in solid-state physics at that time – helped in proving the correctness of DMRG. These methods also led to a more general view of the problem [3], resulting in many algorithms that have potential applications in various problems, in particular solving QUBOs by finding the ground state of a quantum spin glass. We have used the algorithms presented in [4] to solve the models derived in the present manuscript.

Both quantum computers and the mentioned classical algorithms may not provide the energy minimum and the corresponding ground state (as it is not trivial to reach it [5]) but another eigenstate of the problem with an eigenvalue (i.e., a value of the objective function) close to the minimum. The corresponding states are referred to as "excited states." Another important point in interpreting the results of such a solver is the degeneracy of the solution: the possibility of having multiple equivalent optima.

In analyzing these optima, it is helpful that for up to 50 variables, one can calculate the exact ground states and the excited states closest to them using a brute-force search on the spin configurations with GPU-based high-performance computers. In the present work, we also use such algorithms, in particular those introduced in [6] for benchmarking and evaluating our results for smaller examples. This way we can compare the exact spectrum with the results obtained from the D-Wave quantum hardware and the variational algorithms.

SV. FURTHER EXAMPLES OF SOLUTIONS

In this Section we present solutions of dispatching problems depicted in Fig. S1 on railway line line No. 191. Let us first examine the solutions obtained with simple heuristics: with FCFS in Fig. S2 and with FLFS in Fig. S3. It appears

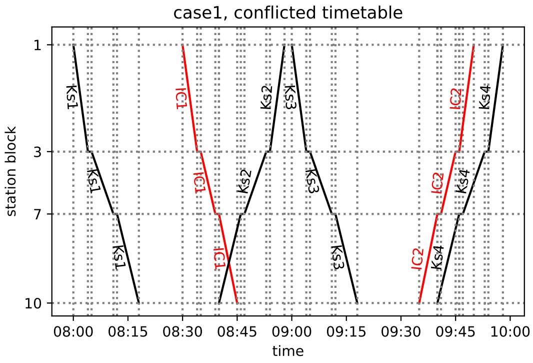

(a) Case 1 – single conflict, observe that the additinal delay of Ks2 will propagate to the delay of Ks3.

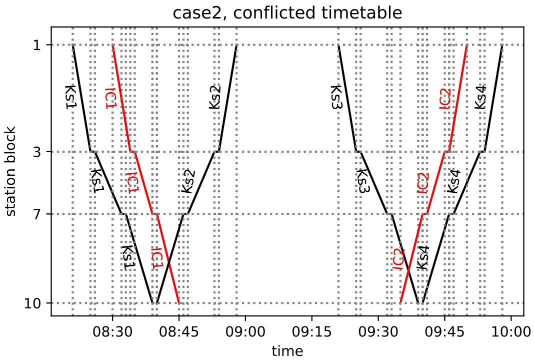

(b) Case 2 – two conflicts, simillar to Fig. 1(a), but with no impact of Ks2 on Ks3.

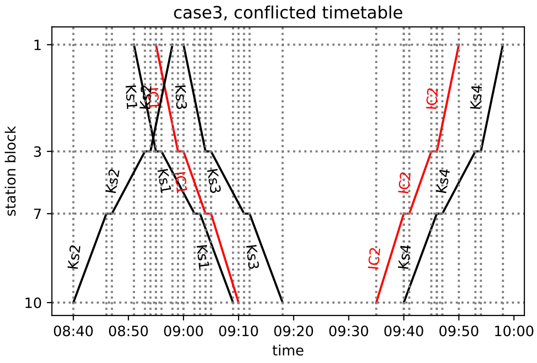

(c) Case 3 – multiple conflicts.

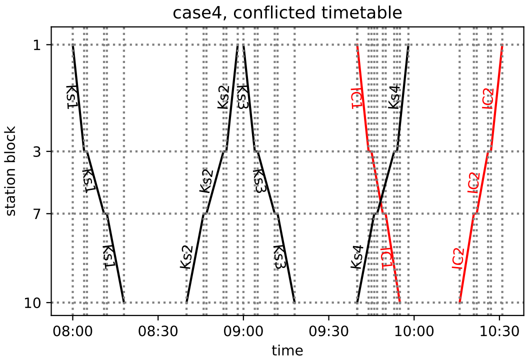

(d) Case 4 – conflict that is straightforward to resolve.

FIG. S1: The conflicted timetables, various types of conflicts.

that these heuristics can yield trouble-causing solutions. This situation suggests a solution in which one train needs to have a time-consuming stopover on a particular station; see Figs. 2(c),3(b). (Such problems sometimes appear in real-life train dispatching too.) Finally, if the problem is easily solvable, as in case 4, all the methods analyzed in the paper give the same solution. This serves as a quality test of our method. It is also interesting to compare these with the result of the AMCC approach in Fig. S4.

Now let us turn our attention to the solutions obtained from our QUBO model in the same situation. The model was solved with CPLEX's QUBO solver (results in Fig. S5) and tensor networks (results in Fig. S6). Recall that this model offers a high flexibility in decisions on train prioritization. It focuses on the train delay propagation on subsequent trains, as illustrated by the comparison of all the solutions of case 1. Provided Ks2 is delayed, an additional delay of Ks3 would happen (which we call a "cascade effect"). Furthermore, the tensor network output in Fig. S6 demonstrates the degeneracy of the ground state and the solutions in the low excited state, which, however, do not have a relevant impact on the dispatching situation. We remark also that all CPLEX solutions in this case coincide with those obtainable from the linear IP model.

[1] J. Lange and F. Werner, J Sched **21**, 191 (2018).
[2] U. Schollwöck, Rev. Mod. Phys. **77**, 259 (2005).
[3] F. Verstraete and J. I. Cirac, Phys. Rev. B **73**, 094423 (2006).
[4] M. M. Rams, M. Mohseni, D. Eppens, K. Jałowiecki, and B. Gardas, Phys. Rev. E **104**, 025308 (2021).
[5] J. Czartowski, K. Szymański, B. Gardas, Y. V. Fyodorov, and K. Życzkowski, Phys. Rev. A **100**, 042326 (2019).
[6] K. Jałowiecki, M. M. Rams, and B. Gardas, Comput. Phys. Commun **260**, 10.1016/j.cpc.2020.107728 (2021).

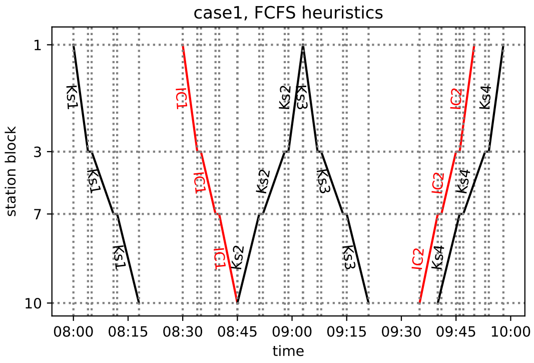

(a) Case 1 – "cascade effect"; the delay of Ks2 causes a further delay of Ks3.

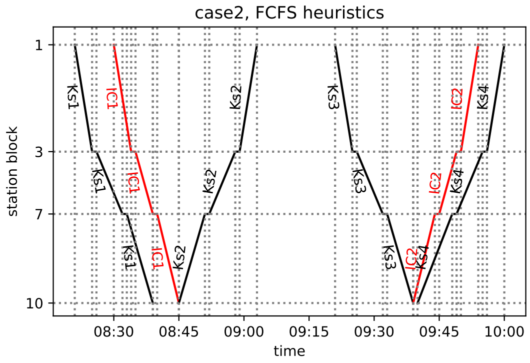

(b) Case 2 – optimal solution reached rather "at random": probably it is reached because the problem is relatively simple.

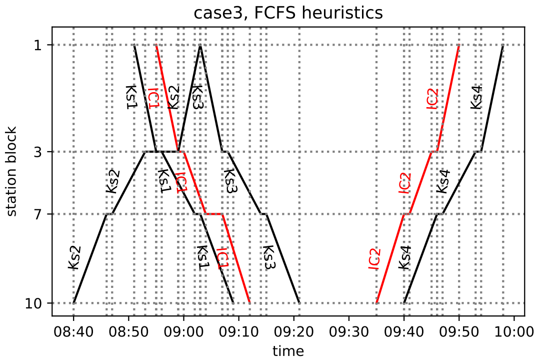

(c) Case 3 – a problematic solution with undesirably long waiting times of certain trains; observe the stopover of Ks2.

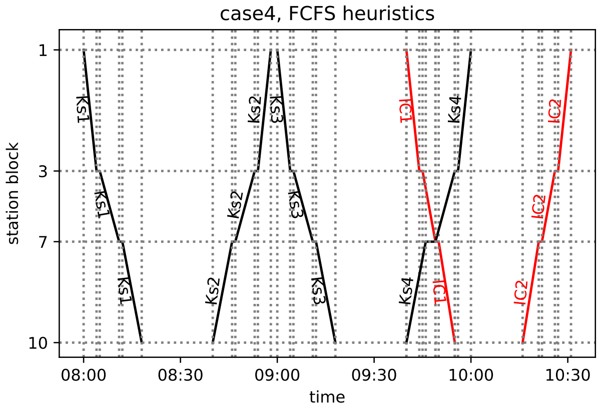

(d) Case 4 – optimal solution according to all methods.

FIG. S2: The FCFS solutions, some with a trouble-causing stopover of a particular train.

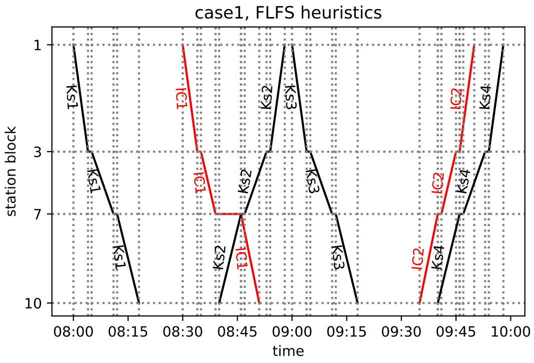

(a) Case 1 – optimal solution is reached "at random," as is its duplicate in Fig. 3(b), which is an undesired solution.

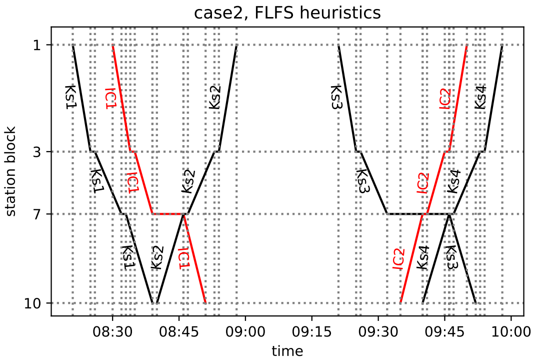

(b) Case 2 – duplicate of the solution in Fig. 3(a) causing an stopover of Ks3.

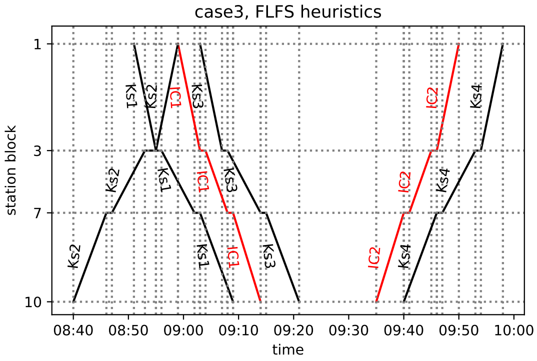

(c) Case 3 – no unacceptable stopovers.

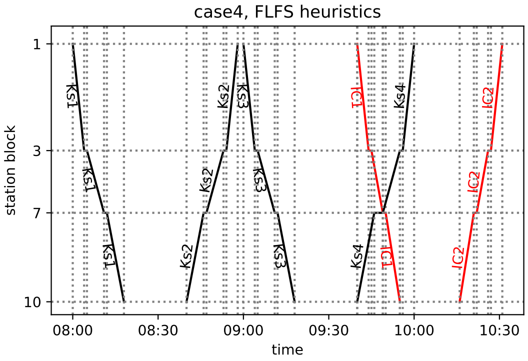

(d) case 4 – optimal solution according to all methods.

FIG. S3: The FLFS solutions, some with a trouble-causing stopover of a particular train.

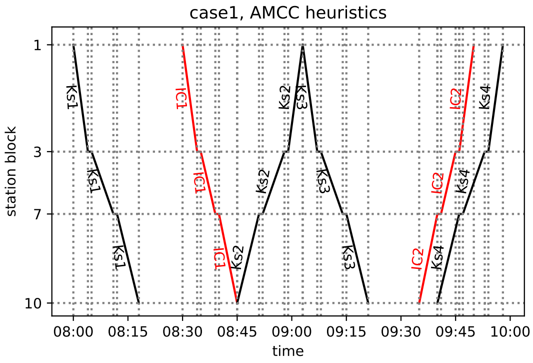

(a) Case 1 – "cascade effect," the delay of Ks2 causes further a delay of Ks3.

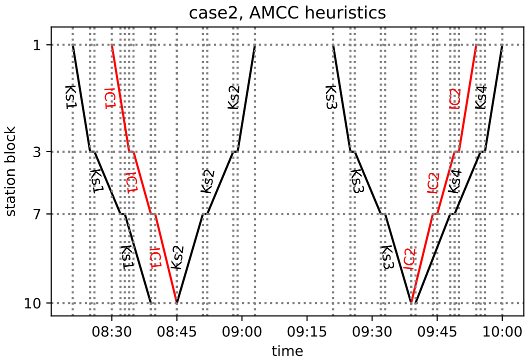

(b) Case 2 – no unacceptable stopovers.

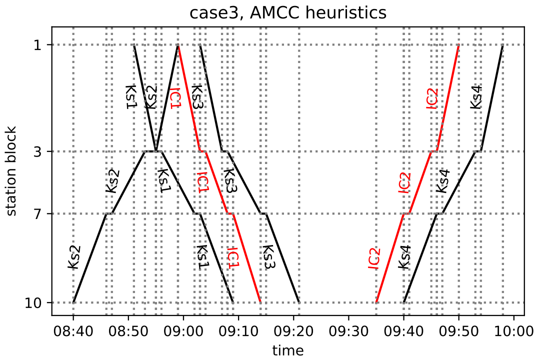

(c) Case 3 – no unacceptable stopovers

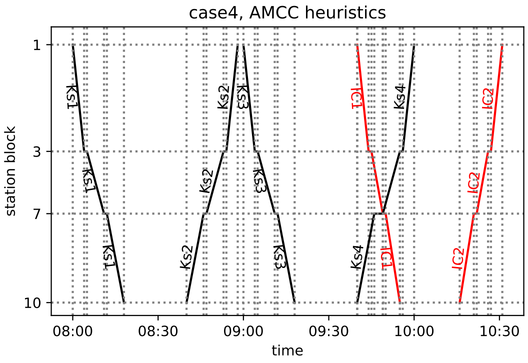

(d) Case 4 – optimal solution according to all methods.

FIG. S4: The AMCC solutions. The minimization of the maximal secondary delays from AMCC excludes unacceptably long stopovers such as those in Figs. 2(c) and 3(b). However, these solutions do not exclude the propagation of smaller delays among several trains ("cascade effect"); see Fig. 4(a).

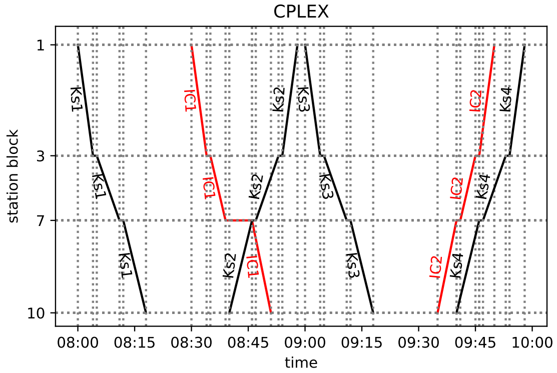

(a) Case 1 – no “cascade effect” (Ks2 does not delay Ks3): a consequence of the pioritization of Ks2.

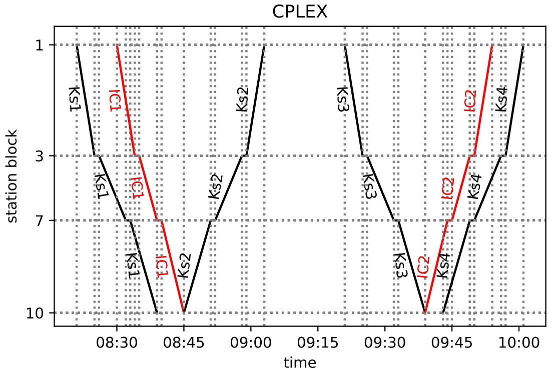

(b) Case 2 – no uncacceptable stopovers.

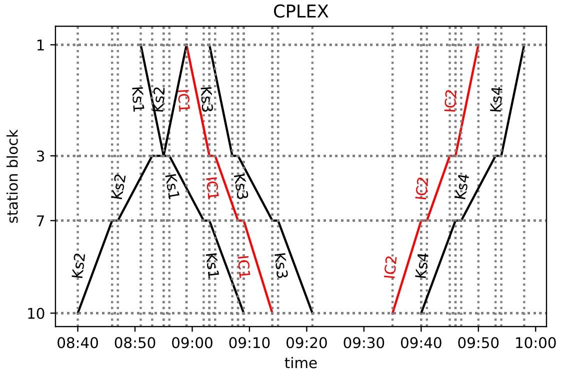

(c) Case 3 – no unacceptable stopovers.

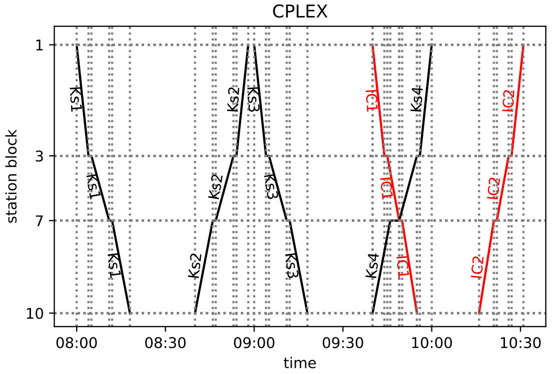

(d) Case 4 – optimal solution according to all methods.

FIG. S5: The CPLEX solutions: exact ground states of the QUBOs. There are no unacceptably long stopovers. Further, the trains' prioritization and the delay propagation to subsequent trains are taken into account. The solutions are the same as these of the linear solver.

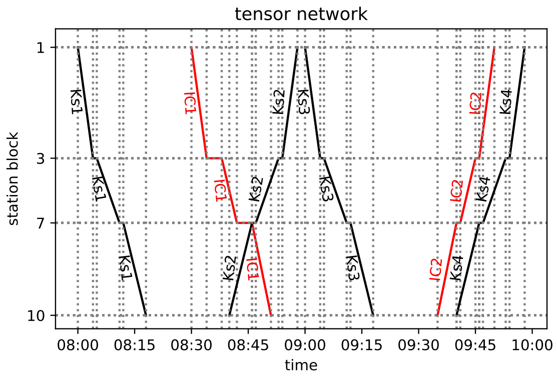

(a) Case 1 – ground state of the QUBO; the degeneracy of the ground state is reflected by a stay of IC1 both at block 3 and at block 7.

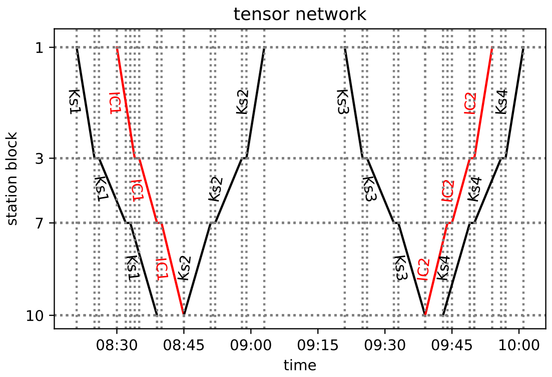

(b) Case 2 – ground state of the QUBO.

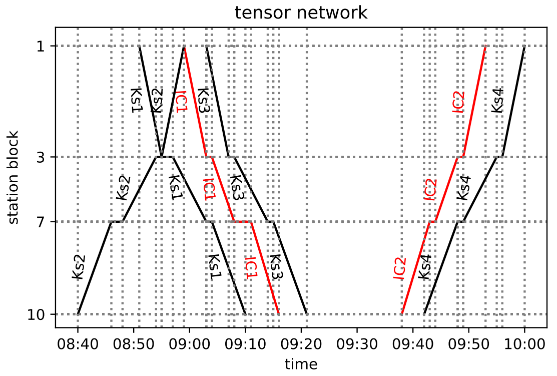

(c) Case 3 – excited state of the QUBO; notice the slightly longer stay of IC1 at block 7.

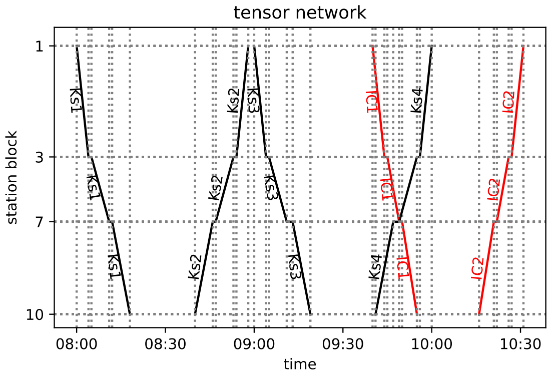

(d) Case 4 – excited state of the QUBO; notice the slightly longer stay of Ks3.

FIG. S6: The tensor network solutions; although the exact ground states were not always achieved, the solutions are equivalent from the dispatching point of view with to in Fig. S5.
